# Impact of the prefabricated forms of NiTi archwires on orthodontic forces delivered to the mandibular dental arch

**DOI:** 10.1186/s40510-021-00385-1

**Published:** 2021-12-01

**Authors:** Akihiko Tachi, Keisuke Tochigi, Naomi Saze, Kazuhito Arai

**Affiliations:** 1 Graduate School of Life Dentistry at Tokyo, The Nippon Dental University, Tokyo, Japan; 2 Department of Orthodontics, School of Life Dentistry at Tokyo, The Nippon Dental University, Tokyo, Japan; 3 Department of Orthodontics, School of Life Dentistry at Tokyo, The Nippon Dental University, 1-9- 20 Fujimi, Chiyoda-ku, Tokyo, 102-8159 Japan

**Keywords:** Preformed archwire, Arch form, Orthodontic force, Archwire classification

## Abstract

**Background:**

Although preformed archwires with a variety of arch forms are currently commercially available, the effects of variation in the shape of these archwires on the orthodontic force at each tooth are not well understood. Therefore, we evaluated the forces delivered by various types of commercially available preformed nickel–titanium alloy (NiTi) archwires in a simulated mandibular dental arch.

**Methods:**

Sixty-three types of 0.019 × 0.025-inch preformed NiTi archwires from 15 manufactures were selected for analysis. The intercanine width (ICW) and intermolar width (IMW) of each archwire were measured at the mean canine and first molar depths of 30 untreated subjects with normal occlusions. Each archwire was placed in a multi-sensor measurement system simulating the mandibular dental arch of subjects with normal occlusions, and orthodontic forces in the facial-lingual direction at the central incisors, canines, and first molars were measured. Correlations between the ICW, IMW, and ICW/IMW ratio of archwires and the delivered forces were analyzed. The archwires were classified into the following four groups according to the ICW and IMW: Control group, ICW and IMW are within the means ± standard deviations of the normal ranges; Ovoid group, narrow ICW and IMW; Tapered group, narrow ICW; and Square group, narrow IMW. The forces were compared among these groups for each tooth.

**Results:**

Significant correlations between the measured archwire width and force to each tooth were found, except between IMW and forces at the central incisors and canines. Significant differences in the forces were found among all groups, except between the Ovoid and Tapered groups at all teeth and between the Ovoid/Tapered and Control groups at the first molar. Significantly greater orthodontic forces in the facial direction were delivered at the central incisors by the archwires in the Ovoid and Tapered groups when compared with the archwires in the other groups.

**Conclusion:**

These findings suggest that there is a possible risk of a clinically significant level of unfavorable orthodontic force being delivered to the mandibular incisors in labial inclination when using a preformed archwire with an ICW that is narrower than the dental arch.

## Introduction

The dental arch form is a key element for achieving stable orthodontic treatment results, and related factors such as occlusal relationship, oral habits, and the positional relationship of the teeth with the basal bone have been widely investigated [1]. In the mandibular dental arch in particular, previous studies have emphasized the importance of preserving the dental arch form and the upright position of the central incisors in relation to the basal bone during orthodontic treatment [1–7]. However, recent studies have observed an expansion tendency in the canine width during the initial stage of orthodontic treatment, even when using light round nickel–titanium alloy (NiTi) archwires [8, 9]. Therefore, in the later stages of treatment, expanded dental arches need to be returned to the pretreatment width, which is often done using rectangular preformed NiTi archwires [10].

When selecting a preformed archwire for a specific patient from the variety of commercially available products, it has been recommended that the arch form matches the pretreatment dental arch form as closely as possible, particular for the mandibular arch, in order to maintain the patient’s original dental arch [11, 12]. However, the results of previous studies that compared the intercanine width (ICW) and intermolar width (IMW) of preformed NiTi archwires with naturally obtained dental arch forms were not in agreement [11, 13–15]. For example, Felton et al. [11] found that only two of 17 kinds of tested preformed archwires closely fit the dental arch forms of 30 subjects with untreated normal occlusions, which covered about 44% of the natural diversity of Class I orthodontic patients in the USA. Also in the USA, Braun et al. [13] compared 16 kinds of preformed archwires for 15 untreated orthodontic patients with Class I dental arch forms using a beta function curve, and they found that the ICW of the preformed archwires was significantly wider than the dental arch form of untreated normal occlusion. Bhowmik et al. [15] also found that 15 kinds of preformed archwires were wider at both the canine and second molar widths than the average dental arches of subjects with normal occlusions in India. In contrast, Oda et al. [14] found that, on average, 20 kinds of preformed archwires available in Japan were significantly narrower than the dental arch form for Japanese subjects with untreated normal occlusions for both the canine and first molar.

Traditionally, dental arch forms have been classified into tapered, square, and ovoid types to characterize each case [16]. In addition to size variations, these classifications are also widely used in the names of currently available preformed archwires [12]. Nevertheless, no standardized objective criteria for these classifications have been established to date [17]. Furthermore, the effect of the application of a preformed archwire that is wider or narrower than the dental arch is simply interpreted as expansion and contraction, respectively, and the magnitude of the delivered force is only estimated based on clinical experience. Therefore, the direction and level of impact of the orthodontic force delivered by the preformed archwires to each tooth in the dental arch remain poorly understood.

Recent in vitro studies have used simulation systems with three-dimensional six-axis force sensors to accurately detect the orthodontic force delivered by preformed archwires to the brackets attached to each tooth of a dental arch [18–20]. These simulation systems have been utilized to evaluate the magnitude and direction of orthodontic forces in three-dimensional space to investigate simulations of cases of labially displaced maxillary canines [18] or lingually displaced mandibular lateral incisors [20].

The purpose of the present study was to evaluate the orthodontic forces delivered by various types of commercially available preformed archwires to the brackets at the central incisors, canines, and first molars in a simulated mandibular dental arch using a multi-sensor, three-dimensional measurement system. The null hypothesis of the present study was that “there is no difference in the orthodontic force delivered to central incisors, canines, and first molars according to the classification of preformed archwire forms.”

## Materials and methods

Means and standard deviations (SDs) of the normal canine depth (4.84 mm ± 0.70 mm) and mean first molar depth (26.66 mm ± 1.57 mm) at the bracket slot (BS) points, which represented the position of the base of the bracket slot of the dental arch form of subjects with normal occlusions, were established based on our previous studies [14, 21]. Means ± SDs as the normal ranges for ICWs (30.18 mm ± 1.32 mm) and IMWs (55.72 mm ± 2.66 mm) of the dental arch form were also established using data from the same previous studies [14, 21].

For the present study, 15 orthodontic suppliers in Japan were contacted from October 2019 to August 2020 to inquire about the availability of 0.019 × 0.025-inch preformed NiTi archwires. Accordingly, sets of 10 or 25 wires were received for 63 types of preformed archwires available from the contacted suppliers (Table 1).

**Table 1:** The 63 types of preformed archwires from 15 manufacturers classified by group

ID	Archwire name	Brand/manufacture	Group
1	Smooth Arch Form	Acme Monaco Inc, New Britain, CT	Control
2	McLaughlin Bennett 5.0 Square-Form	Forestadent, Pforzheim, Germany	
3	Bioform V	G & H Wire Company, Franklin, IN	
4	Europa Form II	G & H Wire Co	
5	Broad Arch Form Large 27 °C Cu-NT	Ormco Corporation, Glendora, CA	
6	Broad Arch Form Large 40 °C Cu-NT	Ormco	
7	Orthos Arch Form Large NT	Ormco	
8	Orthos Arch Form Large 35 °C Cu-NT	Ormco	
9	Orthos Arch Form Large 40 °C Cu-NT	Ormco	
10	Bio-Arch V	TP Orthodontics Inc, La Porte, IN	
11	Ortho Form III	3 M Unitek Orthodontic Products, Monrovia, CA	Ovoid
12	Natural Arch Form III	American Orthodontics, Sheboygan, WI	
13	TANZO Low Force Natural Arch Form III	American Orthodontics	
14	TANZO Mid Force Natural Arch Form III	American Orthodontics	
15	McLaughlin Bennett 5.0 Ovoid-Form	Forestadent	
16	McLaughlin Bennett 5.0 Tapered-Form	Forestadent	
17	Bioform III	G & H Wire Co	
18	Europa Form I	G & H Wire Co	
19	Bio-Arch III	TP Orthodontics	
20	Natural Arch Form II	American Orthodontics	Tapered
21	Euro smile	Forestadent	
22	Bioform II	G & H Wire Co	
23	Trueform II	G & H Wire Co	
24	Orthos Arch Form Small NT	Ormco	
25	Orthos Arch Form Small 35 °C Cu-NT	Ormco	
26	Orthos Arch Form Small 40 °C Cu-NT	Ormco	
27	Bio-Arch II	TP Orthodontics	
28	Straight-Arch II Form	TP Orthodontics	
29	Ortho Form II	3M Unitek	Square
30	Natural arch Form I	American Orthodontics	
31	TANZO Low Force Natural Arch Form I	American Orthodontics	
32	TANZO Mid Force Natural Arch Form I	American Orthodontics	
33	M-flex	Daeseung Medical Co, Seoul, Korea	
34	Accurate	Dentsply Sirona, York, PA	
35	Ideal	Dentsply Sirona	
36	Tynilloy Large	Dentsply Sirona	
37	Tynilloy Small	Dentsply Sirona	
38	Proform	Dynaflex, St. Ann, MO	
39	Straight-Arch Form	Forestadent	
40	Bioform I	G & H Wire Co	
41	Trueform I	G & H Wire Co	
42	Trueform	Lancer Orthodontics, Vista, CA	
43	Broad Arch Form Large NT	Ormco	
44	Broad Arch Form Large 35 °C Cu-NT	Ormco	
45	Broad Arch Form Small NT	Ormco	
46	Broad Arch Form Small 27 °C Cu-NT	Ormco	
47	Broad Arch Form Small 35 °C Cu-NT	Ormco	
48	Broad Arch Form Small 40 °C Cu-NT	Ormco	
49	Tru-Arch Large 27 °C Cu-NT	Ormco	
50	Tru-Arch Large 35 °C Cu-NT	Ormco	
51	Tru-Arch Medium NT	Ormco	
52	Tru-Arch Small NT	Ormco	
53	Tru-Arch Small 27 °C Cu-NT	Ormco	
54	Tru-arch Small 35 °C Cu-NT	Ormco	
55	Pro Form	Ortho Organizers Inc, Carlsbad, CA	
56	Natural arch	Rocky Mountain Orthodontics, Denver, CO	
57	S Arch Form	Shofu Inc, Kyoto, Japan	
58	Accu Form	Tomy international, Tokyo, Japan	
59	Bio-Arch I	TP Orthodontics	
60	Straight-Arch Form	TP Orthodontics	
61	VLP Arch	American Orthodontics	Not classified
62	Bioform IV	G & H Wire Co	
63	Europa form II (Damon)	G & H Wire Co	

**Fig. 1:**
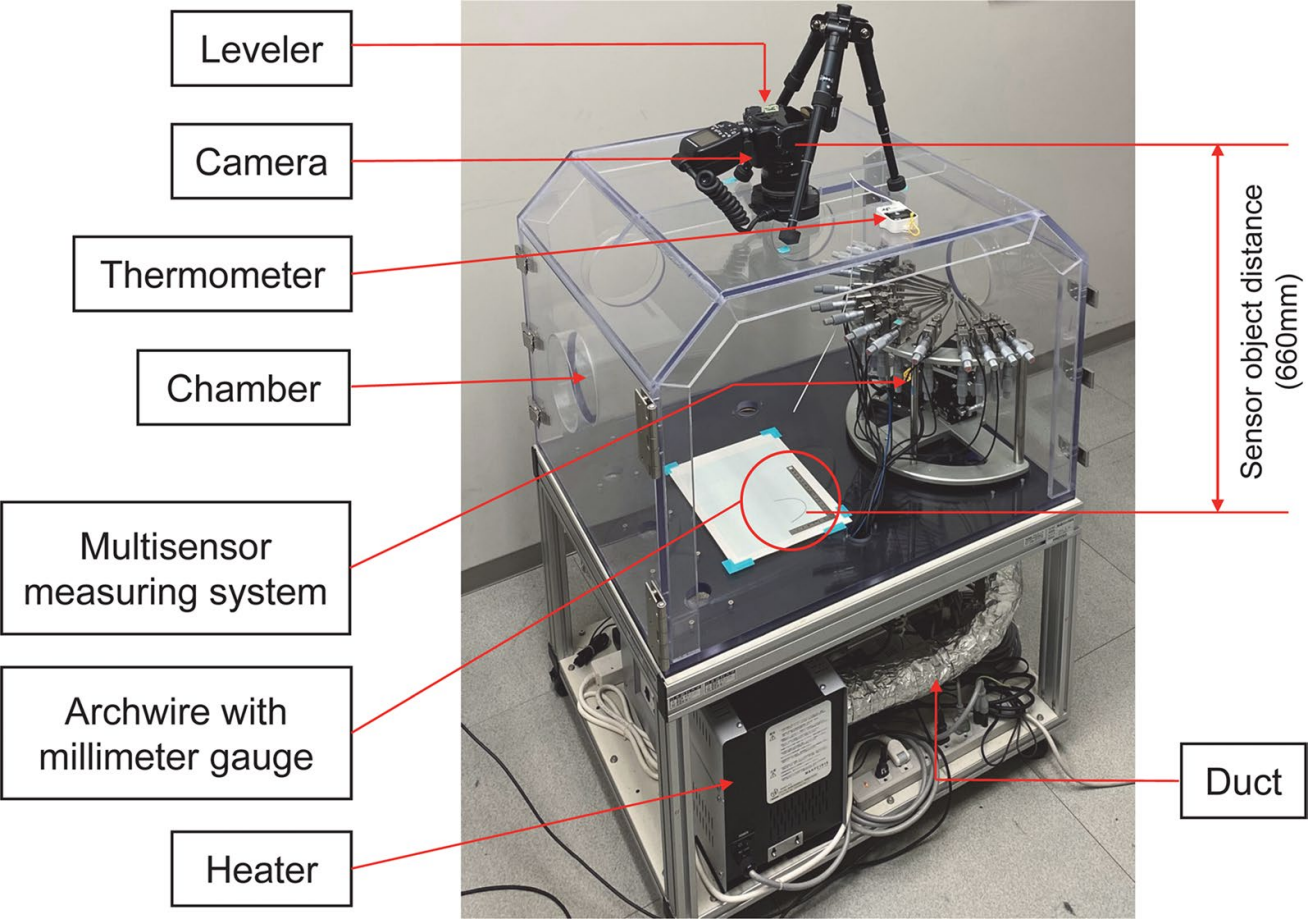
A camera with a macrolens was fixed, using a leveler to obtain standardized images of the archwires. A millimeter gauge was included in the chamber, which was maintained at 37 °C, as monitored by a thermometer

**Fig. 2:**
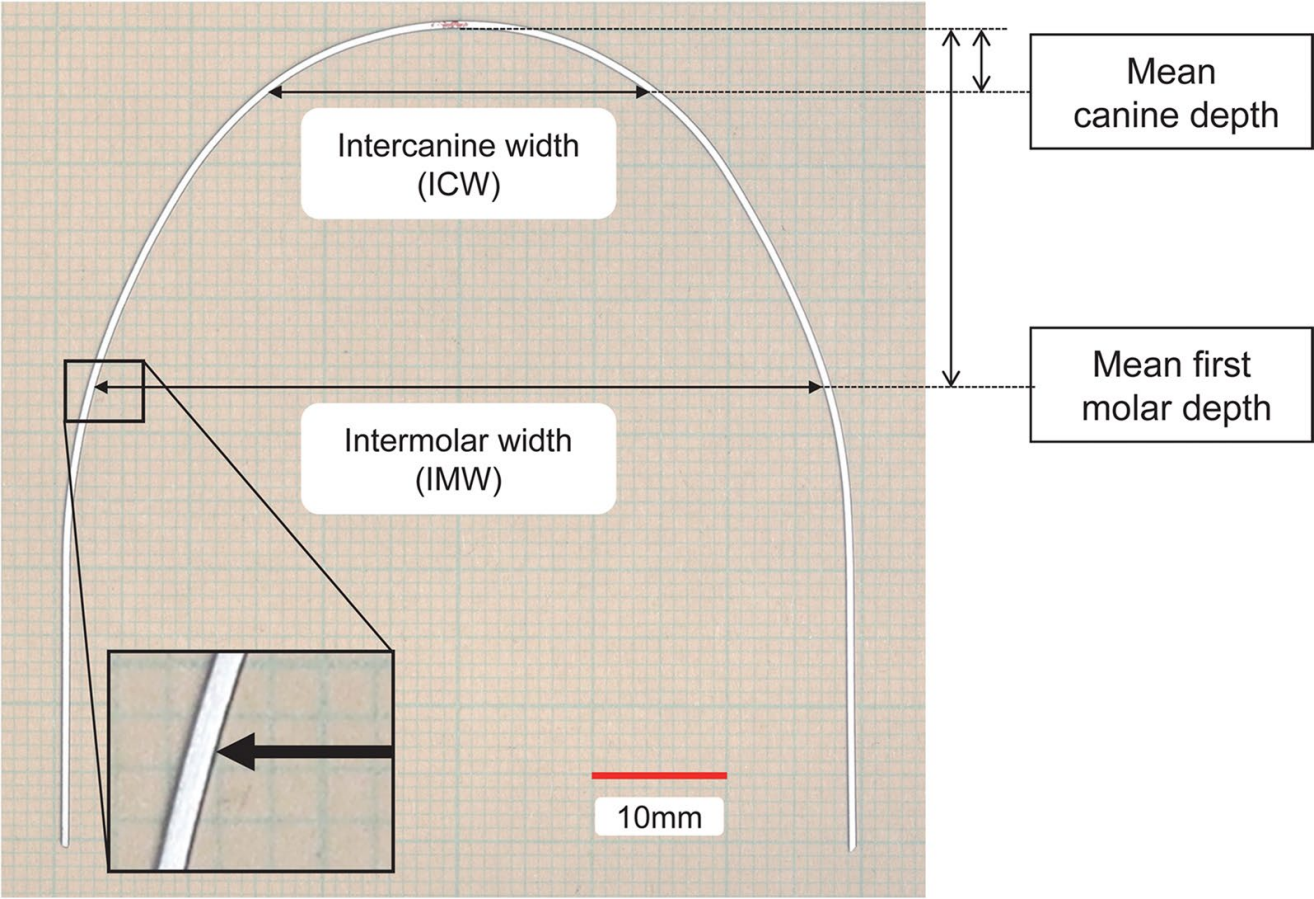
Each archwire was placed on graph paper without fixation, and archwire widths were measured. (example: Orthos Arch Form Small 40 °C Cu-NT, ID: 26)

Two archwires were randomly selected from each set of preformed archwires. A digital camera (EOS Kiss X10, Canon, Tokyo, Japan) with a macrolens (60 mm, F/2.8 Macro EF-S USM, Canon, Tokyo, Japan) was placed above a chamber maintained between 37.0 and 37.5 °C for at least 30 min by a heater, and each archwire was placed in the chamber with a millimeter gauge and without fixation (Fig. 1). A standardized image was then taken at 6000 × 4000 pixels with a resolution of approximately 780 dpi (approximately 0.033 mm/pixel) [21]. The ICW and IMW of each archwire at the normal canine and first molar depths were measured using image analysis software (ImageJ version 1.53e; National Institutes of Health, Bethesda, MD) (Fig. 2) [22].

**Fig. 3:**
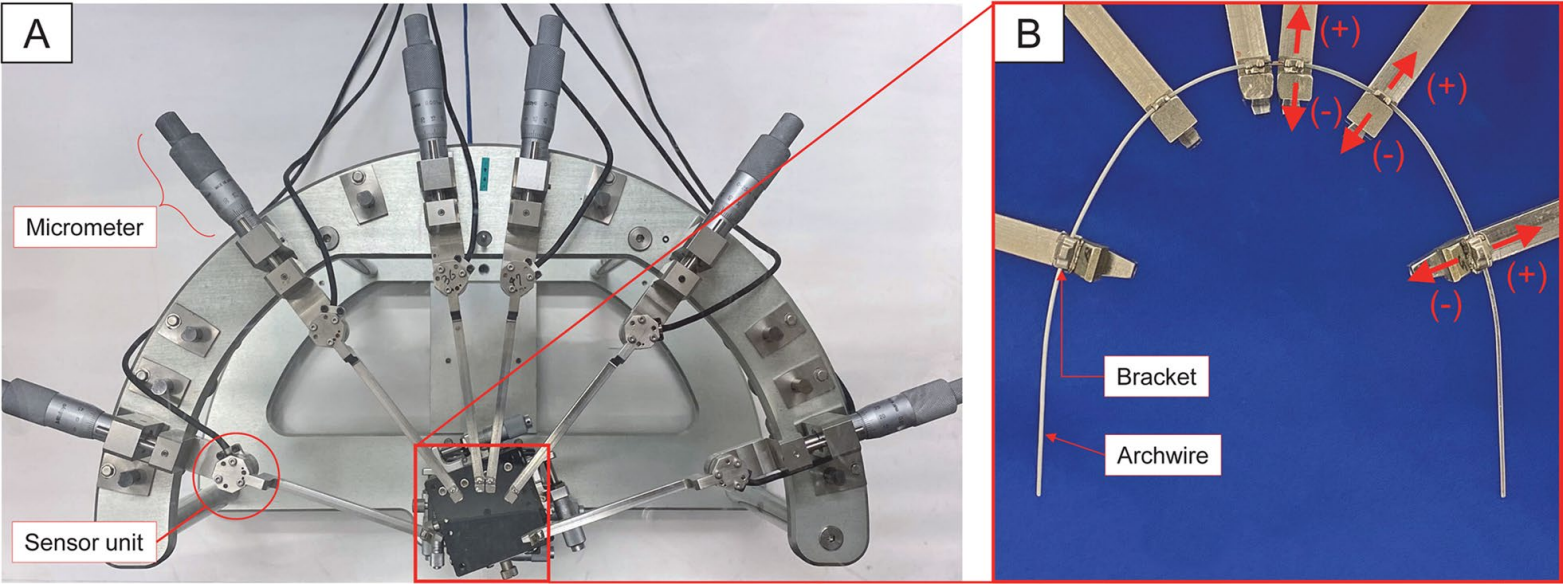
The multi-sensor measurement system. **A** Overview of the multi-sensor measurement system. **B** Close-up view of the stainless steel blocks that simulated the position of the central incisors, canines, and first molars aligned to the dental arch form of normal occlusion. Brackets were placed at the center of the facial surface of each tooth. The orthodontic force in the facial-lingual direction was measured along the perpendicular axis of the center of the bracket slots. When the detected orthodontic force was delivered in the facial direction, a positive value was recorded

A multi-sensor measurement system [19, 20] with six load cells connected to stainless steel blocks that simulate the position of the mandibular bilateral central incisors, canines, and first molars was used to measure the orthodontic forces delivered to the 0.022-inch slot bracket (Damon Q, Ormco Corporation, Glendora, CA). Then, the positions of all stainless steel blocks were adjusted by micrometers (Fig. 3A) until the force delivered to all six brackets reached under 5 gf using a guide wire. Before the measurements, loads were applied to the *x*, *y*, and *z* directions of each load cell using standardized stainless steel weights with 50 gf and 100 gf, and the errors were calculated. The mean value of the error was 2.25%. This value was considered acceptable because it was similar to the accuracy reported by a previous study [18].

A guide wire was fabricated to pass the BS point of the central incisors, lateral incisors, canines, first premolars, second premolars, and first and second molars using a 0.021 × 0.025-inch stainless steel wire (Straight stainless steel rectangular wire, Ormco Corporation).

The inside of the chamber of the measurement system was heated between 37.0 and 37.5 °C for at least 30 min before each measurement and maintained at a constant temperature during each session. Before each measurement, the positions of all brackets on each stainless block were adjusted to fit to the guide wire by micrometers of the measurement system until the force delivered to all six brackets was less than 5 gf. An individual preformed archwire was then ligated to the brackets of the measurement system. The forces delivered to each tooth were measured after stabilizing the temperature inside the chamber between 37.0 and 37.5 °C for at least 10 min. The mean values detected at the right and left sensors were recorded as the force for each tooth type. When the detected force was delivered in the facial direction, a positive value was recorded (Fig. 3B). The mean of two data sets recorded for two archwires randomly selected from each set was calculated and used for analysis as the orthodontic force for the preformed archwire for each tooth type.

Correlations between the arch width measurements (ICW, IMW, and ICW/IMW ratio) and the orthodontic force were analyzed by the Spearman correlation coefficient.

The preformed archwires were classified into the following four groups: (1) Control group, the ICW and IMW were within the normal ranges; (2) Ovoid group, the ICW and IMW were narrower than the normal ranges; (3) Tapered group, the ICW was narrower than the normal range and the IMW was within the normal range; and (4) Square group, the ICW was within the normal range and the IMW was narrower than the normal range.

The Shapiro–Wilk test and the Levene test were performed for each tooth type to confirm the distributions of the data for each preformed archwire group. The results indicated that all measurement data were not normally distributed or homoscedastic; therefore, median and semi-interquartile ranges (SIQRs) of the orthodontic force for each tooth type were calculated for each group. The orthodontic forces were compared between the groups for each tooth type by Kruskal–Wallis and post hoc multiple tests with correction for the false discovery rate [23].

All data were analyzed using SPSS software (version 26.0; IBM, Armonk, NY). The level of significance was set at 5%.

To evaluate intraexaminer error, the measurements were repeated for 10 randomly selected archwires after more than a 2-week interval. The mean intraexaminer error calculated using Dahlberg’s formula [24] was 2.2 gf, which was considered to be within the acceptable range.

To evaluate interexaminer error, another author measured 10 randomly selected archwires. The mean interexaminer error calculated using Dahlberg’s formula [24] was 2.8 gf, which was considered to be within the acceptable range.

## Results

**Fig. 4:**
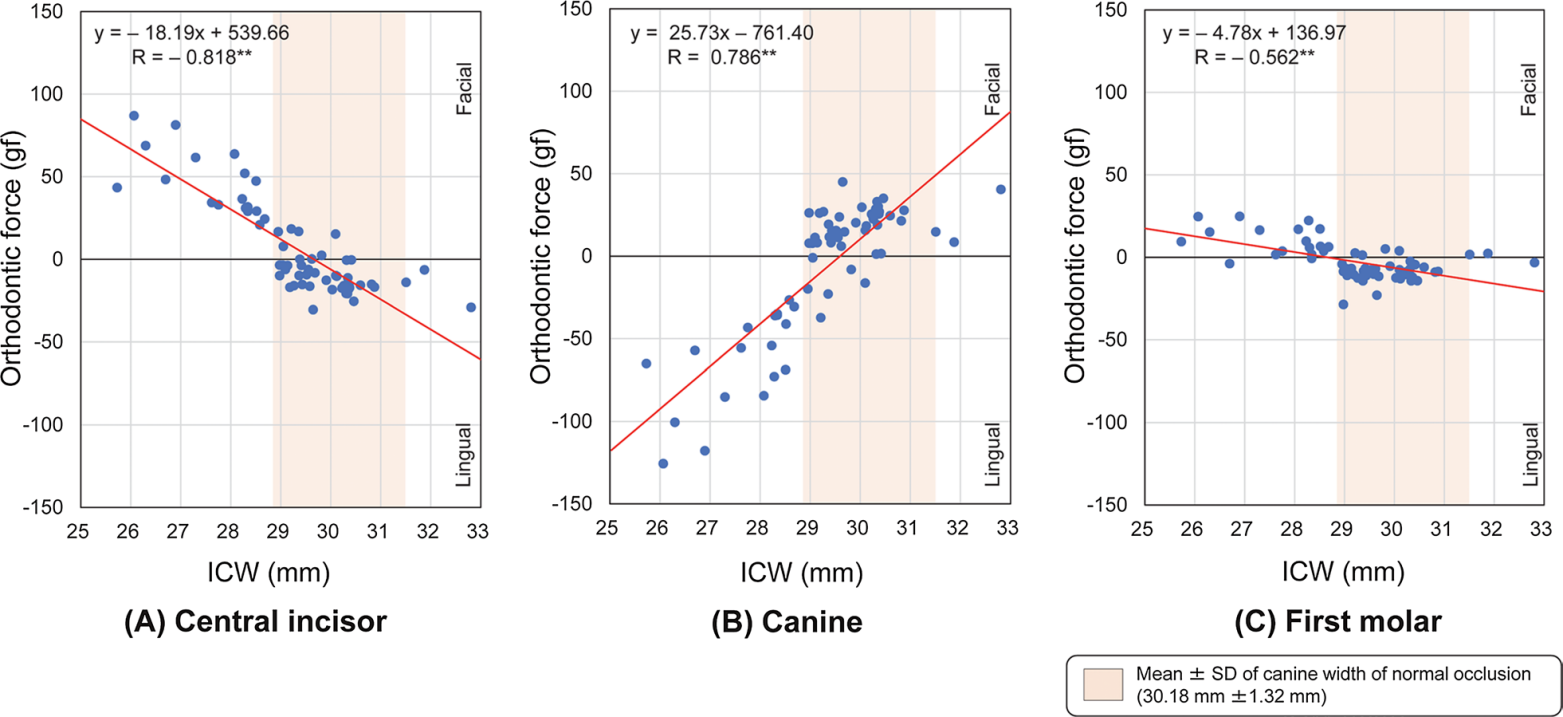
Spearman correlation coefficients between the intercanine width (ICW) and orthodontic forces delivered by 63 types of preformed archwires to the brackets at the **A** central incisors, **B** canines, and **C** first molars. **P < .01 (Facial: positive, Lingual: negative)

Significant negative correlations were observed between the ICW and the orthodontic force delivered at the incisors and first molars (Fig. 4A, C), and a significant positive correlation was observed between the ICW and the orthodontic force delivered at the canines (*P* < 0.01) (Fig. 4B).

**Fig. 5:**
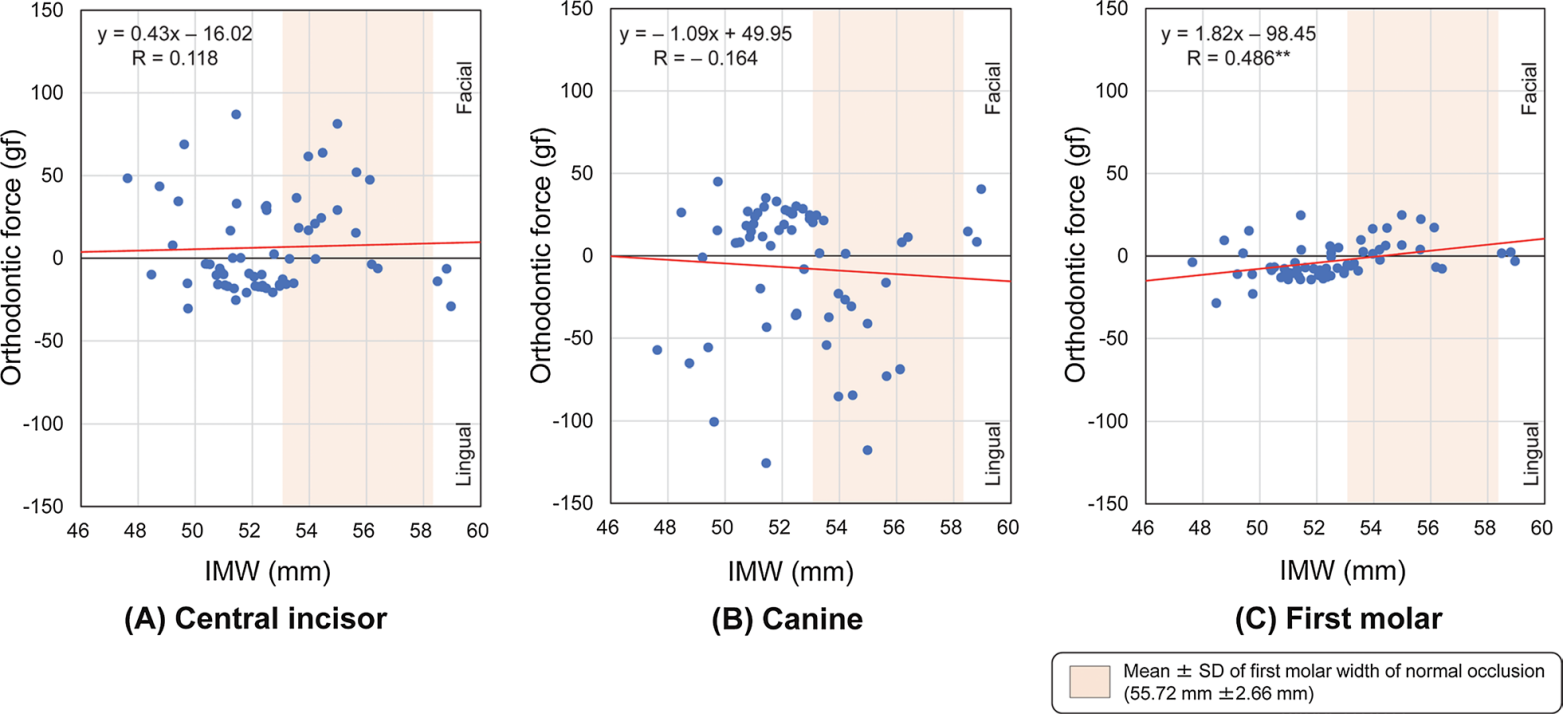
Spearman correlation coefficients between the intermolar width (IMW) and orthodontic force delivered by 63 types of preformed archwires to the brackets at the **A** central incisors, **B** canines, and **C** first molars. **P < .01 (Facial: positive, Lingual: negative)

A significant negative correlation was observed between the IMW and the orthodontic force delivered at the first molars (*P* < 0.01) (Fig. 5C).

**Fig. 6:**
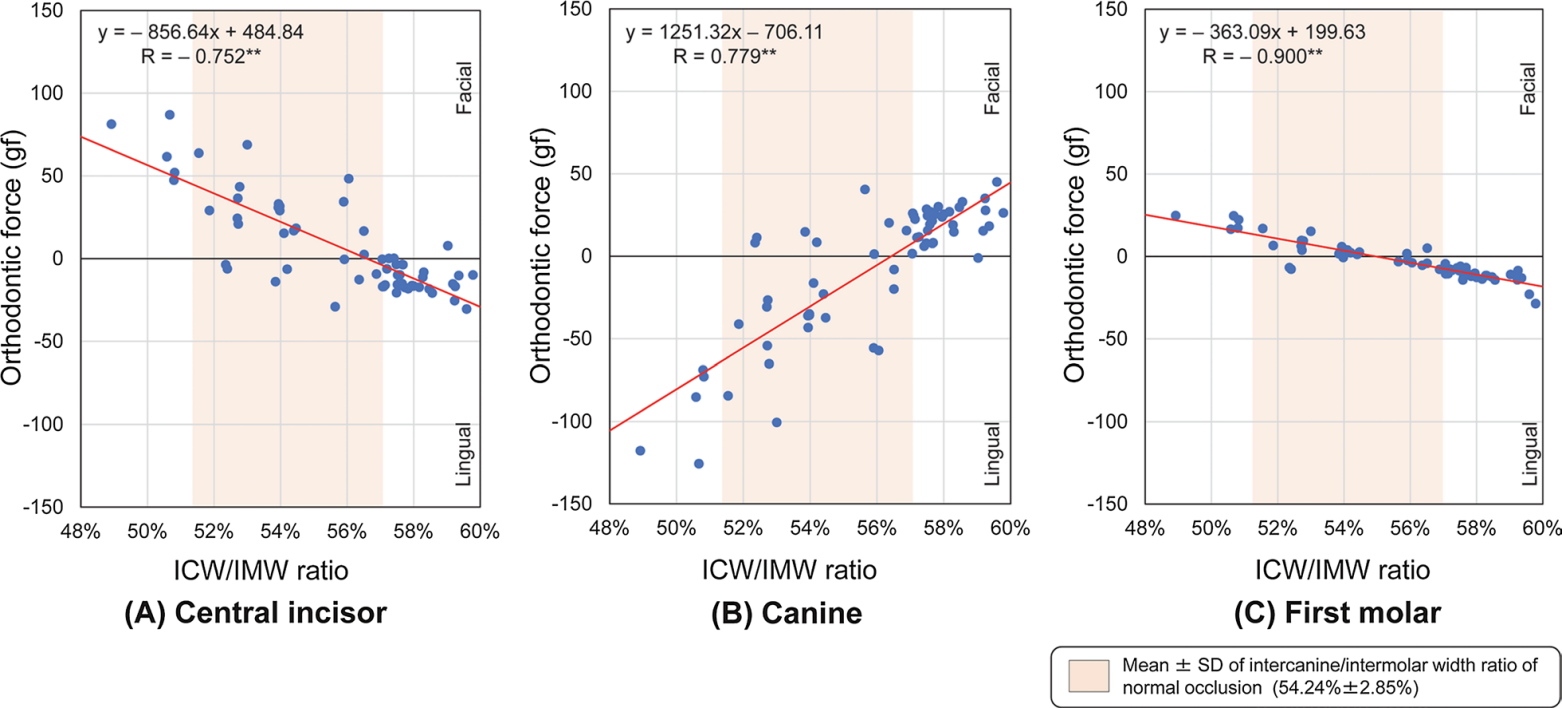
Spearman correlation coefficients between the intercanine/intermolar width (ICW/IMW) ratio and orthodontic force delivered by 63 types of preformed archwires to the brackets at the **A** central incisors, **B** canines, and **C** first molars. **P < .01 (Facial: positive, Lingual: negative)

Significant negative correlations were observed between the ICW/IMW ratio and the orthodontic force delivered at the incisors and first molars (*P* < 0.01) (Fig. 6A, C), and a significant positive correlation was observed between the ICW/IMW ratio and the orthodontic force delivered at the canines (*P* < 0.01) (Fig. 6B).

**Fig. 7:**
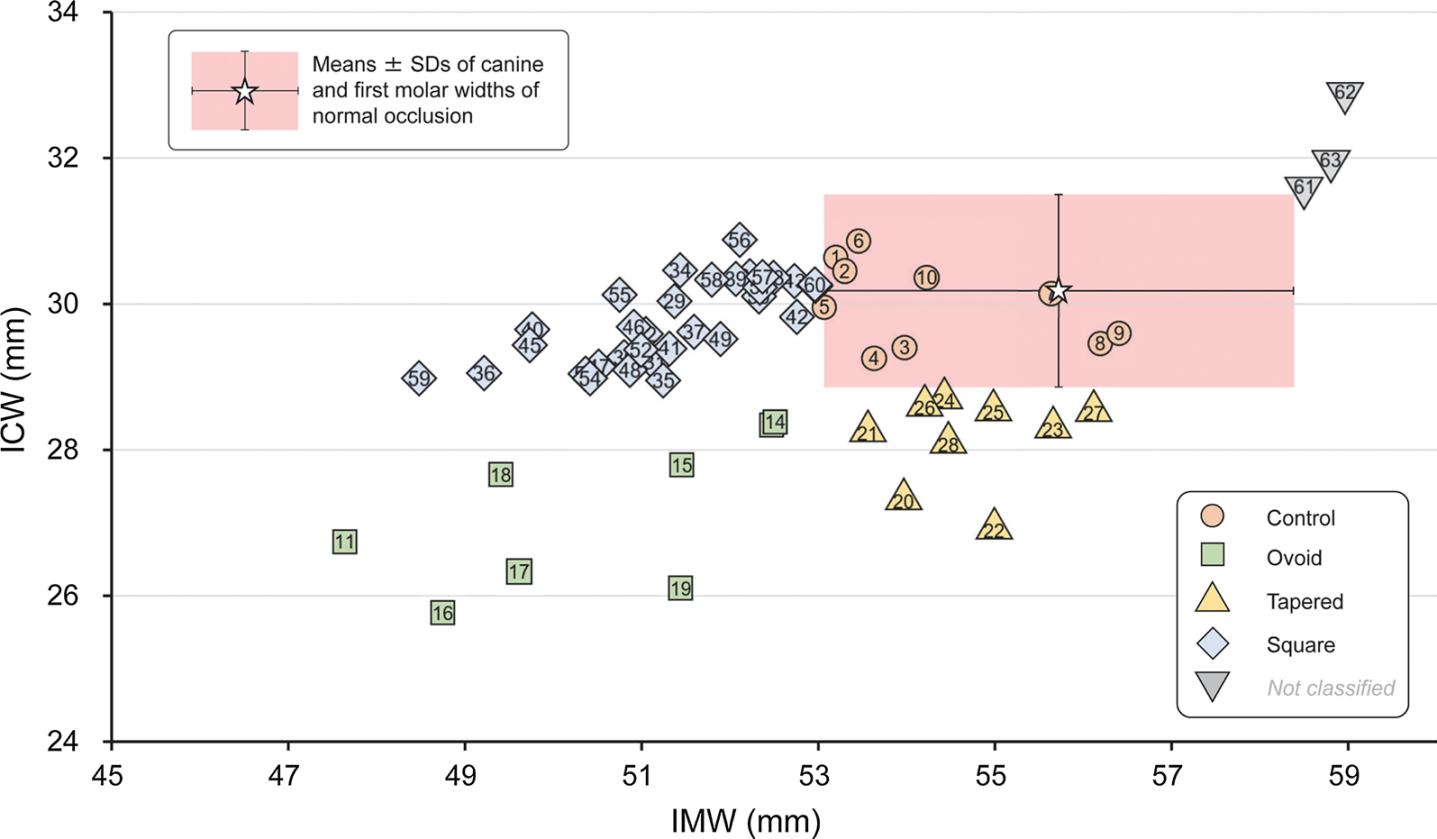
Distribution of 63 types of preformed archwire widths (intercanine width (ICW) and intermolar width (IMW)) with archwire ID numbers and group classifications, and comparison of means and standard deviations (SDs) of the dental arch widths for normal occlusion

The distributions of the ICW and IMW of the preformed archwires in the classified groups were compared with the normal ranges of the ICW and IMW (Fig. 7). Three types of preformed archwires were wider than the normal ranges of the ICW and IMW. These archwires were excluded from the classification because of the small sample size as a group. The remaining 60 types of preformed archwires were classified into the Control group (*n* = 10), Ovoid group (*n* = 9), Tapered group (*n* = 9), and Square group (*n* = 32) (Table 1).

**Fig. 8:**
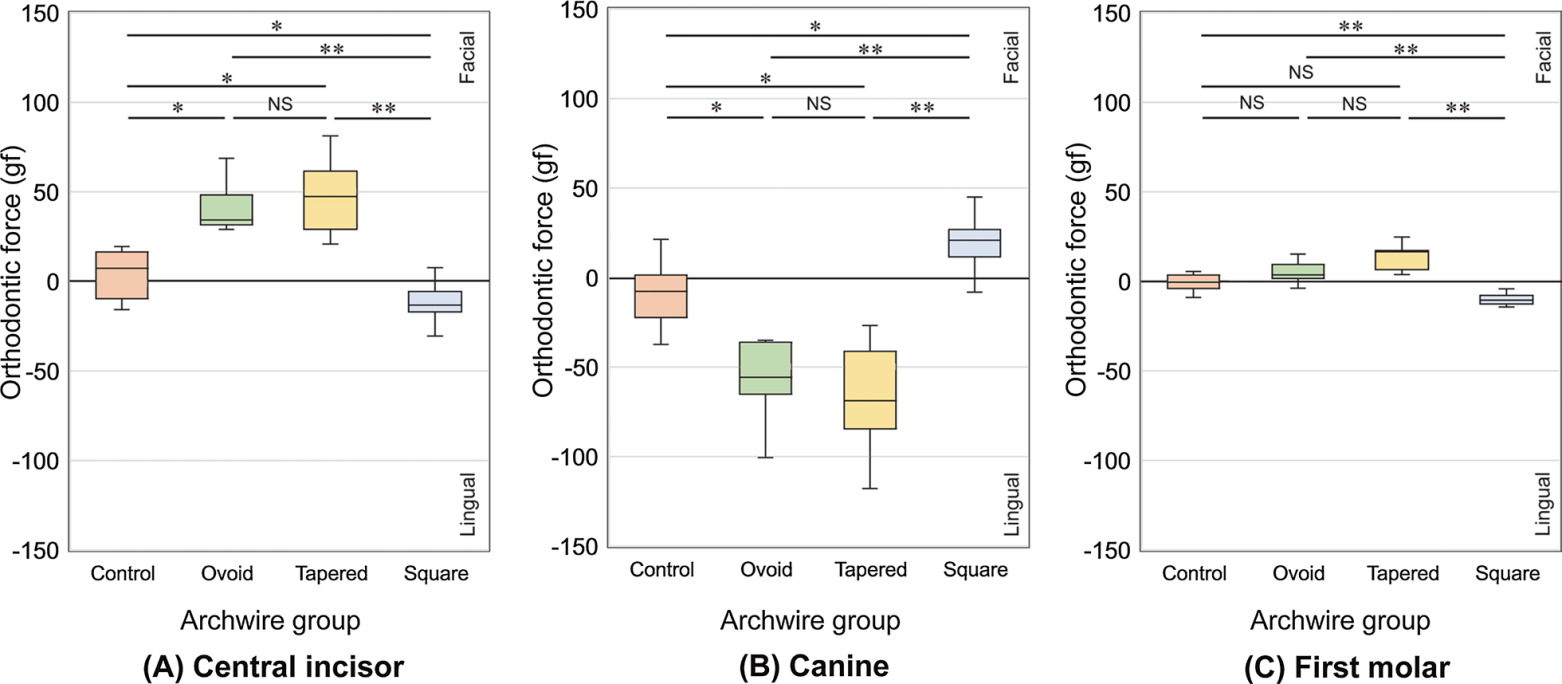
Medians, quantiles, and ranges of orthodontic force measured at the **A** central incisors, **B** canines, and **C** first molars, and comparisons of the four arch form groups by Kruskal–Wallis and post hoc multiple tests with correction for the false discovery rate. *P < .05, **P < .01, NS; Not significant (Facial: positive, Lingual: negative)

At the central incisors, when compared with archwires in the Control group, significantly greater orthodontic force in the facial direction was delivered by archwires in the Ovoid and Tapered groups, and significantly greater orthodontic force in the lingual direction was delivered by archwires in the Square group (*P* < 0.05) (Fig. 8A).

In contrast, at the canines, significantly greater orthodontic force in the lingual direction was delivered by archwires in the Ovoid and Tapered groups, and significantly greater orthodontic force in the facial direction was delivered by the Square group, when compared with archwires in the Control group (*P* < 0.05) (Fig. 8B).

At the first molars, significantly greater orthodontic force in the lingual direction was delivered by archwires in the Square group when compared with archwires in the Control group (*P* < 0.01) (Fig. 8C).

## Discussion

This was the first study to evaluate the influence of shape variation in preformed NiTi archwires on delivered orthodontic forces measured at the central incisors, canines, and first molars aligned along the dental arch of orthodontically untreated subjects with normal occlusions. Significant correlations were observed between the arch width measurements (ICW, IMW, and ICW/IMW) of preformed archwires and the delivered orthodontic forces. After classifying the archwires into Control, Ovoid, Tapered, and Square groups, the delivered orthodontic forces at each tooth were compared among the groups. Significantly greater orthodontic force in the facial direction was delivered at the central incisors by the archwires in the Ovoid and Tapered groups when compared with the archwires in the other groups. These findings suggest that there is a possible risk of unfavorable labial inclination of the mandibular incisors when using a preformed archwire with an ICW that is narrower than the dental arch.

The analyses of correlations between the widths of the preformed archwire and the orthodontic forces delivered to each tooth showed that the wider the ICW or the greater the ICW/IMW of preformed archwires, the stronger the orthodontic force at the central incisor and the first molar in the lingual direction and at the canine in the facial direction. These results suggest that it is possible to mathematically estimate the magnitude of the orthodontic force delivered to each tooth from the ICW or ICW/IMW using the regression equation associated with each correlation coefficient. For example, when a preformed archwire with only 1% greater ICW/IMW is used, increases in orthodontic force of 8.56 gf in the lingual direction, 12.51 gf in the facial direction, and 3.63 gf in the lingual direction were observed at the central incisor, canine, and the first molar, respectively. Similarly, when using a preformed archwire with an ICW that is 2 mm narrower (1 mm on each side) than the patient’s dental arch, increases in orthodontic forces of approximately 36 gf in the facial direction, 51 gf in the lingual direction, and 10 gf in the facial direction were observed at the central incisor, canine, and the first molar, respectively. Previous studies reported that tipping and bodily tooth movement start to occur from 35 g [6] and 50 cN [25]–70 g [6] of orthodontic force, respectively. By comparing these values with the results of the present study, clinically significant orthodontic forces were delivered to the central incisors and canines. A difference (or uncoordination) of approximately 1 mm between the dental arch and the selected archwire is often considered to be within the clinically acceptable limit [26, 27]. Therefore, when preformed archwires that do not fit the dental arch are applied or the selected preformed archwire forms are inconsistent during treatment, an undesirable expansion or contraction force may be delivered, which could possibly increase the risk of round trips [15], root resorption [28], and posttreatment relapse [29].

In contrast, a significant but weak positive correlation was observed between the IMW and the orthodontic force delivered to the first molar. However, the delivered orthodontic force at the first molars was relatively small and may be clinically insignificant. These findings indicate that the variation in the IMW in the preformed archwires was relatively wider than the variation in the ICW, but the influence of the IMW on the orthodontic force was weaker than the influence of the ICW. Therefore, in consideration of the differences in the root surface area for each tooth type, the orthodontic force delivered to the first molars may be limited even when using a thick rectangular preformed NiTi archwire [6]. However, one possible reason for this could be the longer bracket distance between the canine and first molar [30]. Therefore, it is necessary to conduct an evaluation of the forces delivered by preformed archwires that differ from the arch form used in the simulation of a mandibular dental arch that includes premolars.

Although one possible reason may be the influence of differences in the ethnic characteristics of the sampled population [31], the results of previous studies that compared the shapes of preformed archwires and natural dental arch forms have not been in agreement [11, 13–15]. In the present study, the majority of sampled preformed archwires had narrower ICWs and IMWs than the normal dental arch width, which supports the results of previous studies conducted in Japan [14, 21, 22]. In addition, considerable product variation for both the ICW and IMW among the preformed archwires was also noted [11, 13–15, 21, 22]. These findings tend to support previous results that suggested that there is no universally applicable single ideal archwire form for all orthodontic patients [11, 32]. Accordingly, as a new approach, we objectively categorized the sampled preformed archwires into a widely used classification [12, 16] based on the ICW and IMW of the dental arch of subjects with normal occlusions. First, three types of archwires with ICWs and IMWs wider than the normal range of the dental arch of subjects with normal occlusions were excluded because of the small sample size as an independent group. Consequently, 10 types of archwires that were within the normal ranges of the ICW and IMW of the dental arch of subjects with normal occlusions were classified as the Control group. These archwires could be considered the most suitable type for this population [14, 21]. Only nine types were classified into the Ovoid group. The medians of ICW and IMW for the archwires in this group were approximately 1.5 mm and about 2 mm narrower on average than the mean for subjects with normal occlusions; therefore, the archwires in this group could be recommended for female patients [15]. Nine types of archwires within a relatively small range of variation were classified into the Tapered group. Similar to the Ovoid group, few preformed archwires were available for this group. In a previous study, the Tapered type of dental arch was observed in 44% of Caucasian orthodontic patients in the USA and 12% of Japanese orthodontic patients [31]. Therefore, these findings suggest that ethnic background is a factor that contributes to the availability of preformed archwires in specific geographic areas. In contrast, 32 types of preformed archwires from 13 companies were classified into the Square group, which represents the most popular type in Japan. These preformed archwires have been considered imitations of the original “Roth arch form,” which was developed for overcorrection based on clinical experience during the 1970s to compensate for the expansion of the molars during the space closure of extraction cases [6, 14, 33]. However, medical devices should not be designed based only on expert clinical experience or the influence of the market. Therefore, further biomechanical research on preformed archwires is still required to confirm their treatment efficiency.

Compared to the Control and Square groups, the Ovoid and Tapered groups delivered significantly greater orthodontic forces with an average of approximately 40–50 gf in the facial direction, 50–70 gf in the lingual direction, and 5–10 gf in the facial direction at the central incisors, canines, and first molars (Fig. 8). This finding indicates that the application of a preformed archwire with a narrower ICW than the patient’s dental arch would create orthodontic forces that produce labial inclination of the mandibular central incisors and could increase the risk of gingival ressesion [34, 35] and unstable treatment results [1, 2]. In contrast, the archwires in the Square group delivered orthodontic forces that were generally weaker than those in the Ovoid and Tapered groups. When compared with the Control group, orthodontic forces were delivered in the lingual, facial, and lingual directions at the central incisors, canines, and first molars, respectively. However, it should also be noted that a weaker force was delivered by archwires in the Square group for all teeth. As shown in Fig. 7, although the preformed archwires in the Square group had narrower IMWs, the ICWs were within the normal range. Consequently, when the preformed archwires in the Square group were placed in the simulated normal dental arch, the IMWs were increased to the normal width, but the ICWs did not change much and remained close to those of the Control group [21]. In this situation, the orthodontic force delivered to the canines in the facial direction by the archwires in the Square group was still relatively milder than the force delivered in the lingual direction by the archwires in the Tapered or Ovoid groups. Therefore, the Square type preformed archwires, which are the most widely used archwires, might be a relatively safer selection in terms of preventing unfavorable effects on the mandibular incisors and canines [14, 36].

The results of this study should be considered in light of the following limitations. First, in the present study, only the orthodontic force in the facial-lingual direction (*x*-axis) was the focus to investigate the effects on dental arch forms. However, orthodontic forces would also be delivered in the *y*-axis and *z*-axis directions; therefore, additional biomechanical studies in other directions in three-dimensional space and studies evaluating torque must be conducted in the future. Second, the simulated mandibular dental arch was composed of only six teeth without malocclusion in the present study. Since this differed from the clinical situation, more complex action-reaction relationships could be delivered at individual teeth with malocclusion. In the future, it is necessary to simulate the dental arch including lateral incisors and premolars with malocclusion. In addition, the dental arch form set as the normal model in the present study was based on a sample of orthodontically untreated subjects with normal occlusions in a single ethnic group. Since wide individual variation in the dental arch forms of normal subjects has been observed, the influence of orthodontic force created by preformed archwires should be evaluated in orthodontic patient populations with a variety of malocclusions sampled in other geographic areas [7, 37–40]. Third, the characteristics of the nickel–titanium, such as metallurgical structure and structural transformation behavior, would differ among the 15 manufacturers whose products were used in the study. We have not categorized them from this perspective in the present study, but it would be useful to compare the results from this perspective. Finally, because of the nature of the present in vitro simulation study, it was not possible to evaluate the biological reaction of teeth and periodontal tissues, the pressure around the dental arch produced by the occlusion, and the surrounding force exerted by the adjacent muscular tissues of the tongue, lips, and cheeks. Therefore, future research is required to investigate any potential relationships with orthodontic force.

## Conclusions

The null hypothesis was rejected. Within the limitations of the present study, the following conclusions were obtained:

1. Significant correlations between width measurement (ICW, IMW, and ICW/IMW ratio) results for 63 types of sampled commercially available preformed NiTi archwires and the orthodontic forces delivered to the central incisors, canines, and first molars were found, except between the IMW and orthodontic forces delivered to the central incisors and canines.
2. Significant differences in the delivered orthodontic forces were observed among all groups, except between the Ovoid and Tapered groups at all teeth and between the Ovoid/Tapered and Control groups at the first molar.
3. Significantly greater orthodontic force in the facial direction was delivered at the central incisors by the archwires in the Ovoid and Tapered groups compared with the archwires in the other groups.

## Data Availability

Not applicable.

## Abbreviations

ICW: intercanine width
IMW: intermolar width
NiTi: nickel–titanium alloy
SIQR: semi-interquartile range
SD: standard deviation

## References

[1] Graber LW, Vanarsdall RL, Vig KW, Huang GJ (2016). Orthodontics: current principles and techniques.

[2] Tweed CH (1944). Indications for the extraction of teeth in orthodontic procedure. Am J Orthod Oral Surg.

[3] Shapiro PA (1974). Mandibular dental arch form and dimension. Treatment and postretention changes. Am J Orthod.

[4] De la Cruz AR, Sampson P, Little RM, Årtun J, Shapiro PA (1995). Long-term changes in arch form after orthodontic treatment and retention. Am J Orthod Dentofac Orthop.

[5] Burke SP, Silveira AM, Goldsmith LJ, Yancey JM, Van Stewart A, Scarfe WC (1998). A meta-analysis of mandibular intercanine width in treatment and postretention. Angle Orthod.

[6] Proffit WR, Proffit WR, Fields HW, Sarver DM (2014). Chapter 8 biological basis of orthodontic therapy and chapter 9 mechanical principle in orthodontic force control. Contemporary orthodontics.

[7] Ronay V, Miner RM, Will LA, Arai K (2008). Mandibular arch form: the relationship between dental and basal anatomy. Am J Orthod Dentofac Orthop.

[8] Fleming PS, DiBiase AT, Sarri G, Lee RT (2009). Comparison of mandibular arch changes during alignment and leveling with 2 preadjusted edgewise appliances. Am J Orthod Dentofac Orthop.

[9] Nordstrom B, Shoji T, Anderson WC, Fields HW, Beck FM, Kim DG (2018). Comparison of changes in irregularity and transverse width with nickel-titanium and niobium-titanium-tantalum-zirconium archwires during initial orthodontic alignment in adolescents: a double-blind randomized clinical trial. Angle Orthod.

[10] McNamara C, Drage KJ, Sandy JR, Ireland AJ (2010). An evaluation of clinicians' choices when selecting archwires. Eur J Orthod.

[11] Felton JM, Sinclair PM, Jones DL, Alexander RG (1987). A computerized analysis of the shape and stability of mandibular arch form. Am J Orthod Dentofac Orthop.

[12] McLaughlin RP, Bennett JC, Trevisi HJ (2001). Systemized orthodontic treatment mechanics.

[13] Braun S, Hnat WP, Leschinsky R, Legan HL (1999). An evaluation of the shape of some popular nickel titanium alloy preformed arch wires. Am J Orthod Dentofac Orthop.

[14] Oda S, Arai K, Nakahara R (2010). Commercially available archwire forms compared with normal dental arch forms in a Japanese population. Am J Orthod Dentofac Orthop.

[15] Bhowmik SG, Hazare PV, Bhowmik H (2012). Correlation of the arch forms of male and female subjects with those of preformed rectangular nickel-titanium archwires. Am J Orthod Dentofac Orthop.

[16] Chuck GC (1934). Ideal arch form. Angle Orthod.

[17] Arai K, Will LA (2011). Subjective classification and objective analysis of the mandibular dental-arch form of orthodontic patients. Am J Orthod Dentofac Orthop.

[18] Badawi HM, Toogood RW, Carey JP, Heo G, Major PW (2009). Three-dimensional orthodontic force measurements. Am J Orthod Dentofac Orthop.

[19] Tochigi K, Oda S, Arai K (2015). Influences of archwire size and ligation method on the force magnitude delivered by nickel-titanium alloy archwires in a simulation of mandibular right lateral incisor linguoversion. Dent Mater J.

[20] Tochigi K, Saze N, Arai K (2020). Impact of passive self-ligation and conventional elastic ligation on orthodontic force in the simulation of a mandibular lateral incisor linguoversion. Am J Orthod Dentofac Orthop.

[21] Saze N, Arai K (2016). Variation in form of mandibular, light, round, preformed NiTi archwires. Angle Orthod.

[22] Koda T, Saze N, Tochigi K, Arai K (2018). Transverse adjustment of preformed stainless steel archwires to the dental arch form. Orthod Waves.

[23] Benjamini Y, Hochberg Y (1995). Controlling the false discovery rate: a practical and powerful approach to multiple testing. J R Stat Soc Ser B Stat Methodol.

[24] Dahlberg G, Dahlberg G (1940). Errors of estimation. Statistical methods for medical and biological students.

[25] Theodorou CI, Kuijpers-Jagtman AM, Bronkhorst EM, Wagener F (2019). Optimal force magnitude for bodily orthodontic tooth movement with fixed appliances: a systematic review. Am J Orthod Dentofac Orthop.

[26] Camardella LT, Sa M, Guimaraes LC, Vilella BS, Vilella OV (2018). Agreement in the determination of preformed wire shape templates on plaster models and customized digital arch form diagrams on digital models. Am J Orthod Dentofac Orthop.

[27] Haddadpour S, Motamedian SR, Behnaz M, Asefi S, Bagheban AA, Abdi AH (2019). Agreement of the clinician's choice of archwire selection on conventional and virtual models. Angle Orthod.

[28] Eross E, Turk T, Elekdag-Turk S, Cakmak F, Jones AS, Vegh A (2015). Physical properties of root cementum: Part 25. Extent of root resorption after the application of light and heavy buccopalatal jiggling forces for 12 weeks: a microcomputed tomography study. Am J Orthod Dentofac Orthop.

[29] Lucchese A, Manuelli M, Albertini P, Ghislanzoni LH (2019). Transverse and torque dental changes after passive self-ligating fixed therapy: a two-year follow-up study. Am J Orthod Dentofac Orthop.

[30] Adams DM, Powers JM, Asgar K (1987). Effects of brackets and ties on stiffness of an arch wire. Am J Orthod Dentofac Orthop.

[31] Nojima K, McLaughlin RP, Isshiki Y, Sinclair PM (2001). A comparative study of Caucasian and Japanese mandibular clinical arch forms. Angle Orthod.

[32] White LW (1978). Individualized ideal arches. J Clin Orthod.

[33] Roth RH (1987). The straight-wire appliance 17 years later. J Clin Orthod.

[34] Yared KF, Zenobio EG, Pacheco W (2006). Periodontal status of mandibular central incisors after orthodontic proclination in adults. Am J Orthod Dentofac Orthop.

[35] Joss-Vassalli I, Grebenstein C, Topouzelis N, Sculean A, Katsaros C (2010). Orthodontic therapy and gingival recession: a systematic review. Orthod Craniofac Res.

[36] McLaughlin RP, Bennett JC (1995). Bracket placement with the preadjusted appliance. J Clin Orthod.

[37] Ball RL, Miner RM, Will LA, Arai K (2010). Comparison of dental and apical base arch forms in Class II Division 1 and Class I malocclusions. Am J Orthod Dentofac Orthop.

[38] Huth J, Staley RN, Jacobs R, Bigelow H, Jakobsen J (2007). Arch widths in class II-2 adults compared to adults with class II-1 and normal occlusion. Angle Orthod.

[39] Uysal T, Usumez S, Memili B, Sari Z (2005). Dental and alveolar arch widths in normal occlusion and Class III malocclusion. Angle orthod.

[40] Uysal T, Memili B, Usumez S, Sari Z (2005). Dental and alveolar arch widths in normal occlusion, class II division 1 and class II division 2. Angle Orthod.

